# Unexpected Cross-Reaction with *Honigbergiella*-Like DNA in a PCR for Detection of Bovine *Tritrichomonas foetus*

**DOI:** 10.3390/pathogens10040441

**Published:** 2021-04-08

**Authors:** Joanna Dąbrowska, Jacek Karamon, Maciej Kochanowski, Jacek Sroka, Tomasz Cencek

**Affiliations:** Department of Parasitology, National Veterinary Research Institute, Partyzantow Avenue 57, 24-100 Puławy, Poland; j.karamon@piwet.pulawy.pl (J.K.); maciej.kochanowski@piwet.pulawy.pl (M.K.); jacek.sroka@piwet.pulawy.pl (J.S.); tcencek@piwet.pulawy.pl (T.C.)

**Keywords:** *Tritrichomonas foetus*, cattle, PCR, cross-reaction, *Honigbergiella* sp.

## Abstract

The prevalence of bovine *Tritrichomonas foetus* infection has decreased almost to zero in most European countries, such as Poland, which has been *Tritrichomonas foetus*-free since 1997. However, trichomonosis is a notifiable disease and there is a duty to examine samples from cattle. In this study, we present an unexpected cross-reaction with *Honigbergiella*-like DNA in a specimen from a bull. The bovine sample was submitted to the Department of Parasitology National Veterinary Research Institute in Pulawy (NVRI) for confirmatory testing after having been examined at the Regional Veterinary Laboratory, during a routine *T. foetus* diagnosis. Positive results from microscopic observation and cultures were confirmed. Noteworthily, parasites grew on Diamond’s medium only after seven days of incubation, while optimal growth of trichomonads is generally observed after two to four days for this medium. Moreover, by using PCR we obtained positive results for the presence of *T. foetus*. However, sequencing of the amplification product revealed 99.62% identity with *Honigbergiella* sp. Our data suggest that false-positive results may occur in commonly used PCR tests. Thus, unexpected results should be carefully interpreted.

## 1. Introduction

*Tritrichomonas foetus* is a flagellate single-cell parasitic protozoan responsible for trichomonosis, which is a venereal disease in the urogenital tract of cattle [1]. Due to the introduction of artificial insemination, the occurrence of *T. foetus* infection disappeared in most European countries, including Poland, which has been *T. foetus*-free since 1997. Nevertheless, trichomonosis is a notifiable disease and is one of the diseases listed by the World Organization for Animal Health (OIE). Moreover, there is a duty to examine samples from cattle used for artificial insemination and those exported to other countries [2,3]. Due to the unspecified symptoms, it is very difficult to diagnose bovine trichomonosis based on clinical signs only. Therefore, it is necessary to use laboratory techniques. The standard diagnostic methods are microscopic observation (with or without prior cultivation on media) and molecular tests. In the case of microscopy, trichomonads may be viewed on a wet mount or stained slide. In order to increase and support cells growth, *T. foetus* can be cultured in vitro in media, including the most commonly used Diamond medium. However, correct *T. foetus* microscopic identification based only on the morphology of the parasite and its characteristic “rolling motion” also has its limitations, such as relatively low sensitivity and accuracy. Moreover, a lack of specificity can be caused by the presence of other intestinal or coprophilic trichomonad protozoa that are not *T. foetus*, which are present in samples from cattle [4]; therefore various molecular methods have been developed to accurately identify *T. foetus* in samples from cattle. 

The major advantages of molecular tests are their high sensitivity and the possibility of DNA amplification, even from dead cells. However, cross-contamination and carry over of amplicons may occur which can lead to the misinterpretation of results. Moreover, PCR can also amplify fragments from other microorganisms, and thus false-positive results have been noted in diagnoses of bovine trichomonosis [5,6]. 

PCR, according to Felleisen et al. [2], is an important molecular tool for the identification of the DNA of *T. foetus* and its used worldwide, especially in governmental and reference laboratories. Thus, the occurrence of false-positive results is related to the loss of Poland’s status as a country free from trichomonosis.

In this context, we present a case of an unspecific reaction with *Honigbergiella*-like DNA identified during a routine microscopic examination and identified using molecular methods. Therefore, it is important to share the research involved in the diagnosis of trichomonosis, especially in relation to the possible use of non-specific PCR products.

## 2. Results

**Figure 1:**
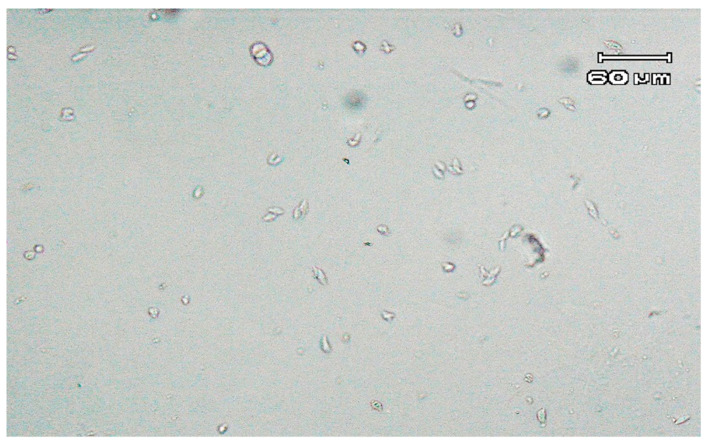
Trophozoites observed under a light microscope (100×, differential interference contrast) in Diamond’s medium after seven days of incubation at 37 °C.

Direct detection of parasites by microscopy revealed the presence of two single trichomonad-shaped microorganisms. Further microscopic observation of the modified Diamond’s medium, conducted between two and seven days, revealed only a few trichomonad-like cells. However, after one week of incubation, examination of the culture showed a higher concentration of forms resembling trichomonads. The majority of the detected parasites had an almond pyriform shape (Figure 1). Moreover, microorganisms exhibited a jerky, rolling motion. 

**Figure 2:**
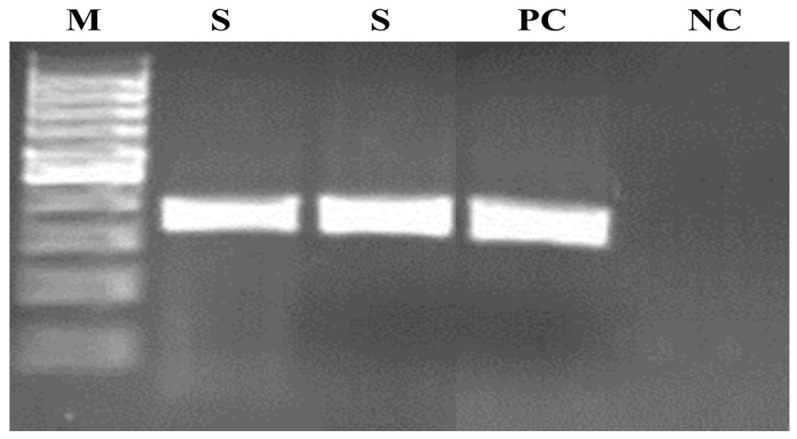
Agarose gel electrophoresis of the PCR product according to Felleisen et al. [2]. Lanes: M—molecular weight marker (100 bp DNA ladder), PC—positive control (DNA of *T. foetus* ATCC (American Type Culture Collection) 30924, NC—negative control, S—examined sample.

Conventional PCR according to Felleisen et al. [2] resulted in the production of a band characteristic of *T. foetus* (347 bp) (Figure 2).

**Figure 3:**
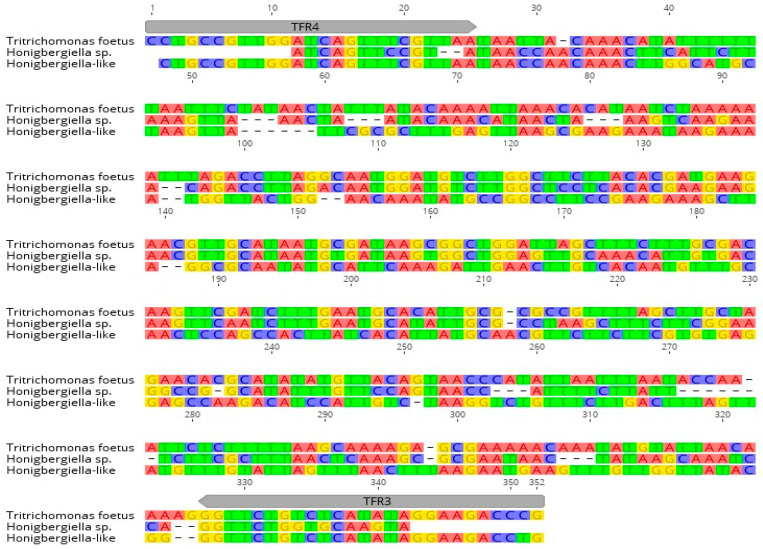
Alignment of *Honigbergiella*-like sequence with sequences of *T. foetus* ATCC 30924 (accession number MW233858) and *Honigbergiella* sp. ATCC 50321 (accession number AY319274.1) found in GenBank™. Localization of primers TFR3 and TFR4 used for PCR are indicated by arrows.

However, based on the results obtained with Sanger sequencing and a subsequent blast analysis, the presence of *T. foetus* in a sample from a bull was excluded. Analysis via BLAST sequence similarity searching revealed that the closest homology of the identified microorganisms (named *Honigbergiella*-like) was with *Honigbergiella* sp. ATCC (American Type Culture Collection) 50321 (GenBank™ accession number AY319274.1), resulting in an identity of 99.62 %. Multiple sequence alignments of *Honigbergiella*-like with *T. foetus* ATCC 30924 and *Honigbergiella* sp. ATCC 50321 sequences taken from GenBank™ were assessed and are shown in Figure 3. The sequence of the detected parasite was submitted to GenBank under accession number MW233858.

## 3. Discussion

Trichomonosis is a venereal disease of cattle and can cause seriously economic losses. Therefore, identification of *T. foetus* in Polish bovine specimens would increase the risk of reintroducing this dangerous parasite into the cattle population. Based on the results from traditional routine microscopic examination with cell culture and PCR, we assumed that we had identified trichomonads. However, sequencing indicated that there were microorganisms other than *T. foetus*. After subsequent blast analysis we found that the closest homology of the identified microorganisms was with *Honigbergiella* sp. ATCC 50321 (GenBank™ accession number AY319274.1) with 99.62% identity. *Honigergiella* sp. is closely related to *T. foetus* because its belongs to the same clad, Parabasalia. However, *Honigbergiella* sp., also known as *Pseudotrichomonas keilini*, is still poorly understood. According to Hampl et al. [7], this microorganism switched recently between a free-living habitat in fresh waters and endobiosis. Similarly, closely related to the *Honigbergiella* sp. Microorganism, *Honigbergiella ruminantium* possibly switched recently from a free-living to a commensal way of life. However, our blast analysis revealed that identity between *Honigbergiella*-like DNA and *H. ruminantium* was 97%. Thus higher identity was found between *Honigbergiella*-like DNA and *Honigbergiella* sp. Furthermore, according to Hampl et al. [7], it is possible that *Honigbergiella* sp. may also be present in feces from cattle. Thus, we cannot exclude the fact that *Honigbergiella*-like is a non-pathogenic commensal. Therefore, it is important to avoid fecal contamination of samples which may be confused with *T. foetus* by removal of material from around the external genitalia of cattle.

The ‘gold standard’ diagnostic method for bovine trichomonosis is a direct microscopic examination and in vitro cultivation in commercial media. However, both methods have limitations, due to the possibility of a false-positive result occurring and the relatively low effectiveness rate of 81–91% for male specimens [4]. In our study, we identified a few microorganisms with the same morphology and characteristic jerky movements as *T. foetus*. Noteworthily, the examination of Diamond’s medium revealed the highest concentration of parasites after seven days of incubation at 37 °C, whereas, according to Lun et al. [8], *T. foetus* should rapidly die after the fifth day in this medium. A probable explanation of this fact is that *Honigbergiella* sp. is closely related to that identified in our study by sequencing *Honigbergiella*-like DNA in optimal growth conditions at 25 °C. Thus, on the basis of on our findings, we assumed that, in the case of *Honigbergiella*-like DNA, the peak concentration is reached later than the maximum growth of *T. foetus*.

Currently, molecular tests are used as a good alternative for the diagnosis of trichomonosis. Among several PCRs developed in the last 25 years, conventional PCR, according to Felleisen et al. [2], has been applied successfully in many laboratories and has high specificity for the detection of trichomonosis. However, to the best of our knowledge, we are the first to identify a cross-reaction in a sample from a bull by conventional PCR [2]. In our survey, we found that TFR3 and TFR4 primers amplified a fragment of *Honigbergiella*-like DNA, although there were several mismatches in the target gene that did not affect the reaction. Similar unspecific results with *T. foetus* in PCR were obtained by Frey et al. [5] where *Simplicimonas*-like DNA was identified in vaginal swabs of female cattle. Nevertheless, in this study, real-time PCR based on detection via fluorescence resonance energy transfer (FRET) was used as a molecular tool for *T. foetus* identification. In this study, the authors received positive results from reactions in 25/34 samples and the signals occurred mostly between cycles 36 and 40, while a few occurred in cycle 33. It is worth noting that the positive control was regularly detected at around cycle 26. Furthermore, the melting curves of the amplification products of the *Simplicimonas*-like DNA were different from the positive control of the reaction. The melting peaks of the reaction with *Simplicimonas*-like DNA appeared at about 59 °C, whereas the positive control had its melting peak at about 65 °C. Moreover, the sequencing analysis showed that the product of the PCR reaction (*Simplicimonas*-like DNA) had a 91% homology to *Simplicimonas* sp. A similar reaction was performed by Schommer et al. [6], where a minor groove-binder DNA probe (TaqMan^®^ MGB) was used, and this reaction also amplified *Simplicimonas* sp. 

## 4. Materials and Methods

### 4.1. Specimen from Cattle

The bovine sample (preputial fluid from a bull) from Kujawsko-Pomorskie province was tested at the laboratory of the Regional Veterinary Laboratory in Bydgoszcz during routine diagnosis for trichomonosis. Microscopic examination and cultivation in Diamond’s medium revealed the presence of forms that resembled *T. foetus*. Therefore, the bovine sample was submitted to the Department of Parasitology NVRI in Pulawy. 

The initial identification of parasites was carried out microscopically on a slide, where 2–4 drops of the specimen were screened for the presence of trichomonads. Then, in order to multiply the parasites, 200 µL of the sample was inoculated into modified Diamond’s medium (MDM) according to the previously described protocol [9,10] and incubated at 37 °C. The development and growth of the parasites were monitored daily (from days 2–8 post-inoculation) by microscopic examination with 100× magnification.

### 4.2. PCR, Sanger Sequencing and Analysis

Tubes containing Diamond’s medium with microorganisms were centrifuged at 400× *g* for 4 min, and 100 μL of sediment was collected. A DNeasy Blood and Tissue Kit (50) (Qiagen, Germany), was used for genomic DNA extraction, according to the manufacturer’s instructions for cultured cells. In the last isolation step, DNA was eluted in 200 μL of buffer. A PCR reaction, according to Felleisen et al. [2], amplified the fragment specific to *T. foetus* (347 bp). PCR amplification was performed in a 50 μL reaction mixture, including 1 pmol of each primer (TRF3 and TRF4); 200 μL of each dNTP (Fermentas); 250U Taq polymerase (Qiagen); 5 μL 10× concentrated PCR buffer (Qiagen); 28.6 μL of DNAse-free water (Fermantas); and 1μL of DNA. As a positive control, DNA isolated from the reference strain of *T. foetus* (ATTC 30924) in distilled water was used as a negative control. PCR analysis was performed with 1 cycle of 94 °C for 30 s, 40 cycles of 67 °C for 30 s, 72 °C for 90 s and a final extension step of 72 °C for 15 min. 

Agarose gel electrophoresis was carried out, followed by 2% agarose staining with ethidium bromide gels using a Mini-SubCell GT chamber and Power Pac Basic (Bio-Rad, Munich, Germany). The *T. foetus*-positive product from the PCR was subjected to bi-directional Sanger sequencing using a commercial sequencing service (Genomed S.A. Company, Warsaw, Poland). The sequenced data were analyzed with Primer3 in Geneious R7 software (Biomatters, http://www.geneious.com, accessed on 16 July 2018). The sequence of the contig was compared in GenBank using BLAST searches. For multiple sequence alignments, CLUSTAL OMEGA (https://www.ebi.ac.uk/Tools/msa/clustalo/, accessed on 1 October 2019) was used. 

## 5. Conclusions

In conclusion, this study revealed the presence of *Honigbergiella*-like microorganisms in a preputial specimen from a bull. Additionally, PCR for *T. foetus* identification also amplified *Honibergiella*-like DNA. Thus, microscopic identification is insufficient, and therefore molecular identification confirmed by sequencing is needed. Moreover, the development and improvement of the specificity of molecular methods against organisms such as *Honibergiella* sp. is important in the field of *T. foetus* diagnosis. 

## Data Availability

The data that support the findings are available in communication or upon reasonable request from the corresponding authors.
